# Response assessment with the CXCR4-directed positron emission tomography tracer [^68^Ga]Pentixafor in a patient with extranodal marginal zone lymphoma of the orbital cavities

**DOI:** 10.1186/s13550-017-0294-z

**Published:** 2017-06-02

**Authors:** Peter Herhaus, Stefan Habringer, Tibor Vag, Katja Steiger, Julia Slotta-Huspenina, Carlos Gerngroß, Benedikt Wiestler, Hans-Jürgen Wester, Markus Schwaiger, Ulrich Keller

**Affiliations:** 1 Internal Medicine III, Technische Universität München, Ismaningerstraße 22, Munich, Germany; 2 Department of Nuclear Medicine, Technische Universität München, Munich, Germany; 3 Institute of Pathology, Technische Universität München, Munich, Germany; 4 Department of Neuroradiology, Technische Universität München, Munich, Germany; 5 Pharmaceutical Radiochemistry, Technische Universität München, Munich, Germany; 6 German Cancer Consortium (DKTK), German Cancer Research Center (DKFZ), Heidelberg, Germany

**Keywords:** Chemokine receptor, CXCR4, Lymphoma, [^68^Ga]Pentixafor, PET, Response assessment

## Abstract

CXCR4 belongs to the family of chemokine receptors. Together with its sole known ligand CXCL12 (SDF-1alpha), it has a pivotal role during organogenesis and for homing of hematopoietic stem cells. CXCR4 is overexpressed in various malignancies, and this is often associated with poor prognosis. Therefore, molecular imaging of CXCR4 bears a great potential for diagnostics and selecting patients for CXCR4-directed therapies. The CXCR4-directed positron emission tomography (PET) tracer [^68^Ga]Pentixafor has been shown to visualize CXCR4 expression in various malignancies in vivo. Whereas this tracer has limitations compared to ^18^F-Fluorodeoxyglucose ([^18^F]FDG) in diagnostic PET imaging in peripheral tumour lesions, it might add valuable information in routine diagnostics and response assessment of tumours in close proximity to the central nervous system (CNS) and malignancies within this organ. As a proof-of-concept, we performed [^68^Ga]Pentixafor PET imaging in a patient with extranodal marginal zone lymphoma (MZL) of the orbital cavities at diagnosis and for post-therapy response assessment. Compared to routinely conducted [^18^F]FDG PET, the lymphoma lesions determined by magnetic resonance imaging (MRI) showed high tracer accumulation at diagnosis, which decreased upon treatment. We therefore propose that imaging of CXCR4 with [^68^Ga]Pentixafor is a potential diagnostic tool for tumours close to or within the CNS and suggest this being studied in clinical trials.

## Main text

### Background

With the emerging role of rational molecular targeted therapies in oncology, molecular in vivo imaging of key targets provides a strong tool as biomarkers for personalized therapeutic options and to refine response assessment. Proof-of-concept visualization of CXCR4 expression with the recently developed CXCR4-directed PET tracer [^68^Ga]Pentixafor has been shown in hematologic malignancies, small cell lung cancer and glioblastoma [1–6].

The chemokine receptor CXCR4 is a 7-transmembrane G protein-coupled receptor with CXCL12 (stromal cell-derived factor-1alpha; SDF-1α) as its sole known ligand. The CXCR4/CXCL12 axis plays an important role during embryonic organogenesis and orchestrates important immunological functions and homing of hematopoietic stem cells to the bone marrow [7, 8]. Besides its physiological role, CXCR4 overexpression has been identified in a variety of human cancers and is regularly associated with poor prognosis.

In B and T cell lymphomas, CXCR4 is constitutively expressed and high CXCR4 levels correlate with poor overall survival. There is growing evidence that in lymphoproliferative disorders, the crosstalk between malignant cells and their surrounding non-malignant microenvironment contributes to tumour growth, metastasis and chemoresistance. CXCR4 expressed by malignant cells and CXCL12 derived from the surrounding stroma play a pivotal role for this interaction [9]. This renders the CXCR4/CXCL12 system a potential aim for future targeted therapeutic strategies.

Herein, we describe for the first time the proof-of-concept of CXCR4-directed PET MRI with [^68^Ga]Pentixafor in response assessment in a patient with extranodal marginal zone lymphoma (MZL) of the orbital cavities and compare it to standard [^18^F]FDG PET imaging.

### Findings

**Fig. 1:**
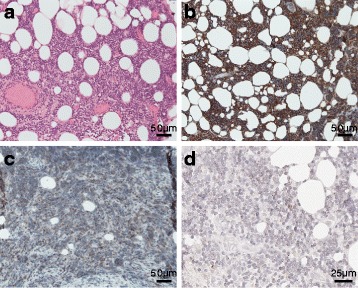
Histology and immunohistochemistry (IHC) for CD20, Bcl-2 and CXCR4 expression in lymphoma biopsy**. a** H&E staining. **b** IHC for CD20. **c** IHC for Bcl-2. **d** IHC for CXCR4

**Fig. 2:**
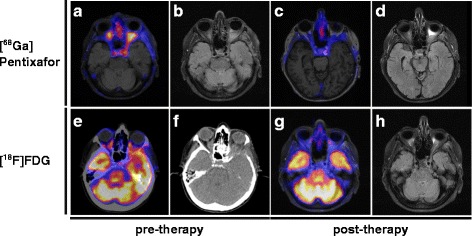
Response assessment with CXCR4-directed PET imaging compared to standard [^18^F]FDG PET. **a**, **c**, **g** PET/MR fusion. **b**, **d**, **h** MR imaging. **e** PET/CT fusion. **f** CT imaging

An 81-year-old female with progressive loss of vision since 6 months was admitted to our hospital. An outpatient ophthalmologist already excluded eye diseases as a direct cause of the symptoms. In the clinical examination, there were no further neurological deficits evaluable and no peripheral lymphadenopathy. B-symptoms were denied. The clinical chemistry and blood count was within the normal range. Cranial MRI revealed bilateral tumourous masses with enhanced contrast agent uptake within the orbital cavities. Pterional trepanation with decompression of the left optic nerve and extended biopsy was performed. Histologically, there was a dense infiltration of CD20+ mature small centrocyte-like, monocytoid and plasmacytoid cells with scattered blasts and few interspersed CD3+ T cells. Morphology and immunphenotype (CD20+, Bcl2+, CD23 partially +, Bcl6−, CD10−, CD5−, Cyclin D1−) was consistent with an extranodular marginal zone lymphoma of mucosa-associated lymphoid tissue (MALT) type. CXCR4 expression was present in approximately 50% of lymphoma cells and showed a perinuclear expression pattern (Fig. 1). In the systemic staging with [^18^F]FDG PET/CT, the lymphoma manifestations within the orbital cavities showed strong uptake (standardized uptake value; SUVmax of 8.7, tumour-to-background ratio (TTB) 4.2) and no further lymphoma lesions were detectable. The PET MRI with the novel CXCR4-directed tracer [^68^Ga]Pentixafor showed specific uptake (SUVmax of 5.9, TTB 2.6) within the CXCR4-positive lymphoma manifestation. Those areas correlated well with the manifestation determined by MRI (Fig. 2a, b, e and f). Compared to [^18^F]FDG PET, the contrast to the surrounding brain tissue was excellent in the CXCR4-directed PET imaging. Taken together, the patient presented with an extranodal MZL restricted to both orbital cavities.

Due to age, comorbidities and patient wish, a treatment with four cycles anti-CD20 antibody (Rituximab 375 mg/m^2^) on a weekly base was conducted. The therapy was well tolerated and vision improved rapidly.

Response assessment with [^18^F]FDG PET and [^68^Ga]Pentixafor PET/MRI was performed 6 weeks after the first administration of rituximab. There was a significant shrinkage of the lymphoma lesions in the standard diagnostic procedure MRI. In [^18^F]FDG PET, there was no specific tracer uptake within the residual lesions determined by MRI (TTB 1.1). Those findings correlated well with negativity for CXCR4-directed PET in the remaining lesions determined by MRI (TTB 1.1) (Fig. 2c, d, g and h). The SUVmax at response assessment was 3.5 for [^68^Ga]Pentixafor and 5.3 for [^18^F]FDG with comparable ΔSUVmax of 2.4 and 3.4, respectively.

### Conclusions

Herein, we describe for the first time that response assessment with the new CXCR4-directed PET tracer [^68^Ga]Pentixafor is feasible and comparable to [^18^F]FDG PET.

Molecular imaging allows visualization of molecular and biochemical processes within the human body. The best-studied PET tracer for in vivo metabolic activity is [^18^F]FDG. This tracer is widely used for diagnostic purposes of metabolic activities in cancer. Several studies over the past decade have established the incorporation of [^18^F]FDG PET in the staging and final response assessment of [^18^F]FDG-avid lymphomas [10]. Adjustment of therapy intensity based on the results of [^18^F]FDG PET interim staging is of special interest, e.g. for reducing chemotherapy-related toxicities, but is currently not used in clinical routine in non-Hodgkins’ lymphomas due.

Molecular imaging further illustrates its role as a biomarker in personalized medicine. In this context, peptides used for imaging can be labelled with a therapeutic radionuclide [11]. In neuroendocrine cancers, in vivo imaging of somatostatin is already used to select patients for peptide-receptor radiotherapy (PRRT) and treatment of this disease with [^177^Lu]Dotatate has shown impressive results [12].

The early experiences with [^68^Ga]Pentixafor show that it has its biggest potential as a biomarker for future PRRT approaches, and we are currently developing trials to evaluate its therapeutic potential in hematologic malignancies. In tumours within or close to the central nervous system (CNS), however, due to its better contrast characteristics compared to [^18^F]FDG PET, the CXCR4-directed tracer [^68^Ga]Pentixafor may improve the current response criteria provided by the International Primary CNS Lymphoma Collaborative Group. In cases where slight abnormalities reside in the MRI, this classification has an uncertainty defining complete remission [13]. We therefore think that negativity for CXCR4 in PET imaging might add valuable information in such cases and should be studied in a prospective trial.

### Methods

#### Synthesis of [^68^Ga]Pentixafor

Synthesis of [^68^Ga]Pentixafor was performed on a fully automated, GMP-compliant procedure using GRP module (Scintomics GmbH, Germany) equipped with disposable single-use cassette kits following the previously described method [5, 6]. Prior to injection, tracer quality was assessed according to the standards of the European Pharmacopoeia available at www.edqm.eu.

#### PET imaging protocols

Pre- and post-therapeutic [^68^Ga]Pentixafor PET was performed on a PET/MRI device (Siemens Biograph mMR, Siemens Medical Solutions, Germany). Injected activity was 148 MBq (pre-therapeutic acquisition) and 110 MBq (post-therapeutic acquisition). PET scans were performed 55 and 40 min after i.v. injection of the tracer (15 min acquisition time, 1 bed position, 3D mode).

A coronal two-point Dixon 3D volumetric interpolated examination (VIBE) T1w sequence was performed for generation of attenuation maps. Diagnostic sequences included axial FLAIR and pre/postcontrast T1w images.

Pre-therapeutic [^18^F]FDG PET for staging purpose was performed on a Sensation 64 Biograph PET/CT scanner (Siemens, Erlangen, Germany) with injected activity of 340 MBq (delay 60 min p.i).

The CT scan protocol included a low-dose CT (26mAS, 120 kV, 5 mm slice thickness) from the skull to the mid-thigh for attenuation correction followed by the PET scan.

The PET scan was acquired in 3D mode with an acquisition time of 3 min per bed position.

Post-therapeutic [^18^F]FDG PET was performed on the aforementioned PET/MRI device 81 min post-injection of the tracer (activity 180 MBq). Acquired diagnostic sequences were analogous to the aforementioned PET/MR protocol.

## Abbreviations

[^18^F]FDG: ^18^F-Fluorodeoxyglucose
CNS: Central nervous system
MALT: Mucosa-associated lymphoid tissue
MRI: Magnetic resonance imaging
MZL: Marginal zone lymphoma
PET: Positron emission tomography
PRRT: Peptide-receptor radiotherapy
SDF-1α: Stromal cell-derived factor-1alpha
SUV: Standardized uptake value
TTB: Tumour-to-background ratio
